# ACCT is a fast and accessible automatic cell counting tool using machine learning for 2D image segmentation

**DOI:** 10.1038/s41598-023-34943-w

**Published:** 2023-05-22

**Authors:** Theodore J. Kataras, Tyler J. Jang, Jeffrey Koury, Hina Singh, Dominic Fok, Marcus Kaul

**Affiliations:** 1 Graduate Program of Genetics, Genomics and Bioinformatics, University of California, Riverside, Riverside, CA 92521 USA; 2 School of Medicine, Division of Biomedical Sciences, University of California, Riverside, Riverside, CA 92521 USA

**Keywords:** Image processing, Machine learning, Cell biology, Computational biology and bioinformatics, Neuroscience

## Abstract

Counting cells is a cornerstone of tracking disease progression in neuroscience. A common approach for this process is having trained researchers individually select and count cells within an image, which is not only difficult to standardize but also very time-consuming. While tools exist to automatically count cells in images, the accuracy and accessibility of such tools can be improved. Thus, we introduce a novel tool ACCT: Automatic Cell Counting with Trainable Weka Segmentation which allows for flexible automatic cell counting via object segmentation after user-driven training. ACCT is demonstrated with a comparative analysis of publicly available images of neurons and an in-house dataset of immunofluorescence-stained microglia cells. For comparison, both datasets were manually counted to demonstrate the applicability of ACCT as an accessible means to automatically quantify cells in a precise manner without the need for computing clusters or advanced data preparation.

## Introduction

Quantifying cells in immunofluorescent images has long been a limiting step in both time and required effort for the analysis of microscopy data used in research. These selective image analysis techniques can provide valuable physiological information and manual counts by trained professionals have been held up as the “gold standard” for quantification^1,2^.

Here we used multiple separate observers’ complete manual counts for comparison to an automatic cell counting methodology. Traditionally, an important aspect of maintaining consistency in cell quantification has been ensuring that a dataset is counted by a single observer who strives for accuracy and reproducibility while ideally being blinded to the experimental conditions. This massively limits the speed at which cell counting data can be processed, as increases in manpower do not always translate to increased speed. Manual counting can struggle with reproducibility and consistency across a dataset due to human error and fatigue. Such issues can be avoided by utilizing computational models which remain consistent over any number of images.

For that purpose we introduce here ACCT: Automatic Cell Counting with Trainable Weka Segmentation (TWS) hosted on GitHub at https://github.com/tkataras/Automatic-Cell-counting-with-TWS.git. TWS provides a machine learning basis for our accessible automatic cell counting methodology, with additional image processing potential provided by scripts in ImageJ, Python, and BeanShell^3,4^. The TWS program provides a graphical user interface (GUI) for training and applying a machine learning classifier that differentiates between cell and non-cell pixels, which are then grouped into cell objects and counted. ACCT is built around this pixel segmentation to provide quantitative validation at the cellular level and assist in optimal classifier selection and application (Fig. 1). ACCT processes single-channel images provided by users. Images with multiple channels can be analyzed using image copies showing one channel at a time and processing image sets for each channel separately.

Two datasets are used in this study to demonstrate performance in varied imaging contexts. The first dataset used is comprised of imaged microglia in mice with and without immune-and-inflammation-activating conditions brought on by the transgenic expression of the envelope protein gp120 of human immunodeficiency virus-1 (HIV-1)^5^. This model of NeuroHIV (HIVgp120tg mouse) provides an observable outcome from the manual counts, an increase in microglia in the presence of HIVgp120 (referred to hereafter as Activated) versus the absence of the viral protein (non-transgenic littermate control, referred to as Resting). ACCT was used to assess the difference in microglia cell numbers from images represented in Fig. 2. For an automatic counting methodology to be effective in an experimental context, it must be able to accommodate the variability in data presentation resultant from experimental conditions^6^. Microglia are known to undergo morphological changes during activation that alter their morphology and appearance when imaged through immunofluorescent staining^7,8^. We focus on a dataset of images of cells immunofluorescence-labeled for ionized calcium-binding adaptor protein-1 (Iba-1) which is a cell type-specific marker and enables visualizing microglia. However, the methodology and accompanying scripts allow for automatic quantification of cells in a wide array of imaging contexts.

The second dataset used is a publicly available set of images of monosynaptic retrograde tracer stained neurons at 200x magnification (Fig. 3). This dataset, which we refer to as the Fluocell dataset, was used in the generation of novel additions to the U-net neural network for cell segmentation^9,10^. Thus, our study tests the validity of ACCT against another published dataset.

### Accessibility

The existence of software tools for use in the life sciences does not inherently lead to an improvement in function^11^. The prerequisite technical knowledge to operate new software tools effectively can create barriers to novel methodologies based on their accessibility. The goal of ACCT is to reduce the barrier to entry for the execution of full semi-supervised imaging studies. ACCT provides the tools to leverage user expertise in handcrafting training data, while providing quantitative tools to efficiently assess training accuracy from a variety of approaches. By reducing the programming knowledge required from users with GUI elements, ACCT increases accessibility of automatic cell counting. Additionally, ACCT performs statistical analysis from the counted images, reducing the technical workload and additionally increasing the tool’s accessibility.

### Related works

There are many ways to address an image segmentation problem. This complex problem centers on assigning an appropriate label for every pixel in an image. The TWS program we utilize is just one of several software tools, including machine learning implementations such as Ilastik^12^ and neural nets like U-Net, ResUNet, and c-ResUnet^9,13,14^.

We have chosen to work with TWS^4^ over Ilastik^12^ due to the increased breadth of default available features, as well as the integration with Fiji and ImageJ that streamlines automated image processing and analysis. This integration with ImageJ made TWS more accessible to build upon for this and future automatic imaging tools.

While programs like TWS and Ilastik provide excellent pixel segmentation with an accessible interface, there is an additional need to assess accuracy and performance at the cell level, rather than the pixel level. ACCT provides a framework for users to accomplish this with minimal file manipulation at the command line. Ilastik’s segmentation does not test models against a validation stage following training of their machine learning model, which increases the risk of overfitting to the training dataset. Thus, we compare the performance of Ilastik to ACCT in our study.

Additionally, we compare the performance of ACCT against CellProfiler, which is a tool commonly used for image analysis which allows users to create modular pipelines^15^. This tool provides pixel level segmentation, although it does not provide automated cell counting with machine learning models without its companion tool CellProfiler Analyst^16^. However, CellProfiler Analyst requires the users to manually modify text and database files in SQL, which requires user knowledge of code editors. For this reason, we do not compare against CellProfiler Analyst.

Finally, ResUNet is a convolutional neural net approach (CNN) to image segmentation and exists as a general tool for image labeling. It was demonstrated to make effective use of training data to make accurate cell segmentation on images with a large variance in the number of cells, as well as the presence of non-cell artifacts. This is a development on U-Net, which has proven effective at bulk cell counting tasks in a variety of contexts. Further, c-ResUnet is an extension of ResUNet^9^. However, CNN models require high processing power to generate results in a reasonable amount of time, which may require accessing expensive computational centers. ACCT is designed to be efficiently functional on commercially available consumer laptops and computers.

## Methods

**Figure 1:**
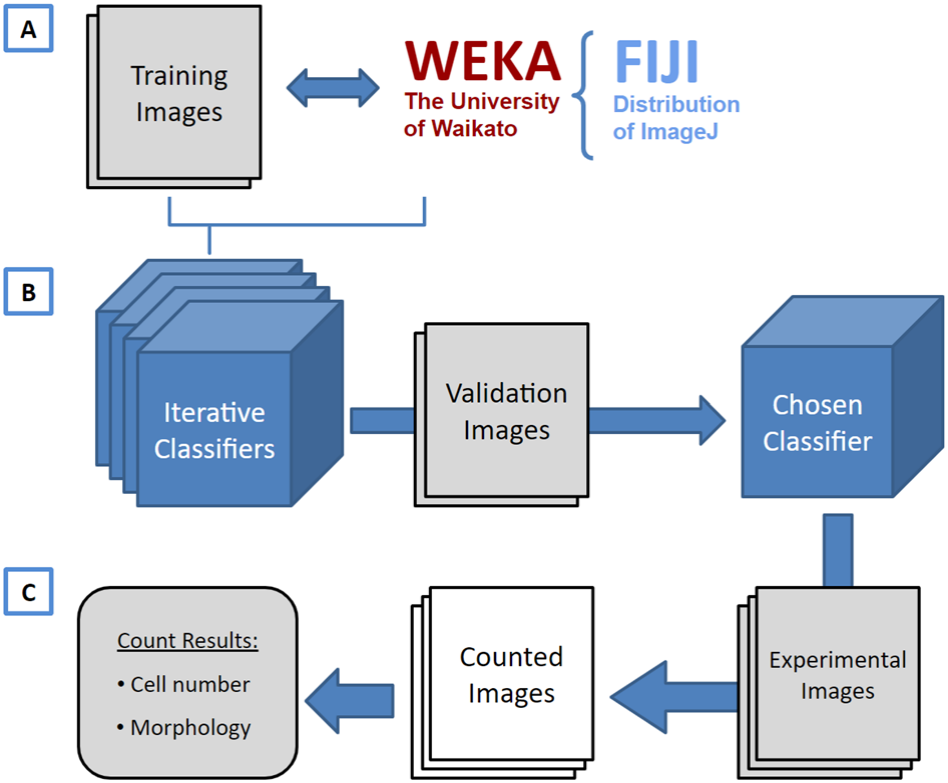
A visual overview of ACCT components and process. (**A**) Weka and a set of training images are employed to create multiple classifiers through iterative training. (**B**) These classifiers are then evaluated in bulk against validation images and the best classifier is chosen by the user. (**C**) The chosen classifier is applied to the experimental dataset for cell quantification, producing a set of counted images and the number of cells in each image, as well as information about the cell morphology. This information includes the area, position, minimum and maximum intensity, circularity, skew, and more details about each cell, available on GitHub in the data availability section.

### Iba-1 Microglia dataset

A dataset comprised of images of Iba-1 positive microglial cells was generated following procedures recently published by our group^17^. In brief, the dataset was derived from brain sections of a model for HIV-induced brain injury (HIVgp120tg), which expresses soluble gp120 envelope protein in astrocytes under the control of a modified GFAP promoter^5^. The mice were in a mixed C57BL/6.129/SJL genetic background, and two genotypes of 9 month old male mice were selected: wild type controls (Resting, n = 3) and transgenic littermates (HIVgp120tg, Activated, n = 3). No randomization was performed. HIVgp120tg mice show among other hallmarks of human HIV neuropathology an increase in microglia numbers which indicates activation of the cells compared to non-transgenic littermate controls^17^. All experimental procedures and protocols involving animals were performed in compliance with National Institutes of Health (NIH) guidelines and approved by the Institutional Animal Care and Use Committees (IACUC) of the Sanford Burnham Prebys Medical Discovery Institute (SBP), The Scripps Research Institute (TSRI), and the University of California Riverside (UCR). The study follows ARRIVE guidelines.

**Figure 2:**
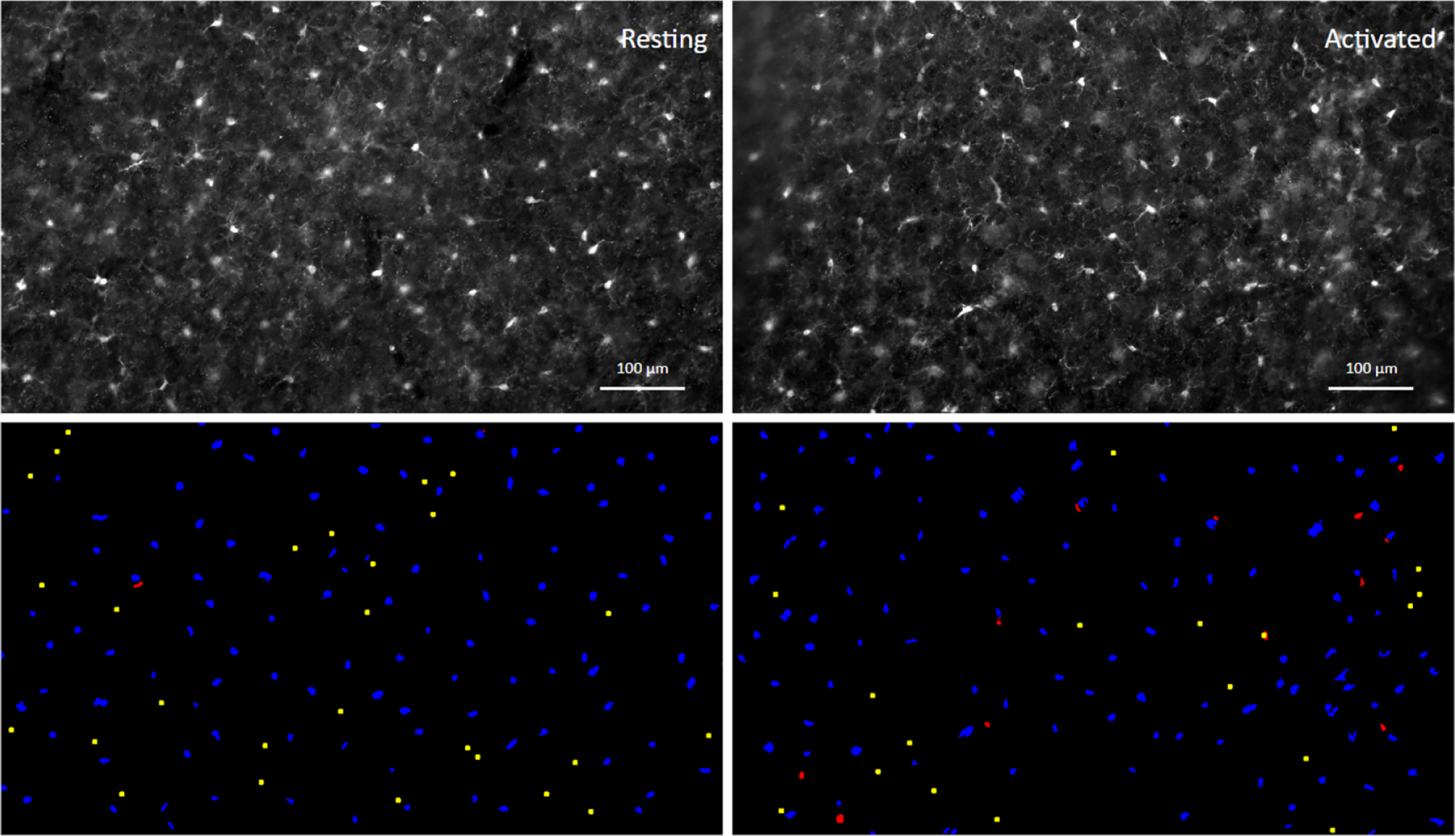
Images of immunofluorescence-labeled microglia before and after segmentation. An example of processed paired images of Iba-1 immunolabeled microglia in cerebral cortex (layer III; Upper panel) of wild-type, non-transgenic (‘Resting’) and of HIVgp120tg mice (‘Activated’), and the accompanying final segmentation images generated via ACCT (lower panel). Resulting object segmentations are color-coded (blue = true positive, red = false positive, yellow = false negative). Segmented objects from images in the same field of view were projected with a size exclusion minimum of 50 pixels for counting. Immunofluorescence staining and acquisition of images are described in previous publications and the Methods Section^17^. Scale bar: 100 $\mu m$.

The procedures for brain tissue harvest, immunofluorescence staining, and microscopy of microglia have been described in a recent publication by our group^17^. In brief, mice were terminally anesthetized with isoflurane and transcardially perfused with $0.9\%$ saline. The mouse brains were removed and fixed for 72 hours at $4^{\circ }\hbox {C}$ in $4\%$ paraformaldehyde^17^. Brain sections were obtained using a vibratome (Leica VT1000S, Leica Biosystems, Buffalo Grove, IL) and cerebral cortex in 40 $\upmu \hbox {m}$ thick sagittal sections spaced 320 $\upmu \hbox {m}$ apart medial to lateral from brains of each genotype. Staining was performed with rabbit anti-ionized calcium-binding adaptor molecule 1 (Iba-1) IgG (1:125; Wako) with secondary antibody Fluorescein isothiocyanate (FITC). For quantification of Iba-1 stained microglia, cell bodies were counted in the cerebral cortex from three fields of view for three sections each per animal. Between 2 and 3 images were collected per field of view to capture as many cells as possible in sufficient focus for identification. Microscopy was performed with a Zeiss 200 M fluorescence deconvolution microscope with a computer-controlled 3D stage and FITC filter. All images were collected using Slidebook software (version 6, Intelligent Imaging Innovations, Inc., Denver, CO). Images were acquired at 10X magnification and pixel resolution 1280x1280 and cropped to 1280x733 pixel area to exclude irregular tissue edges. Representative examples are shown in Fig. 2.

#### Manual counts

Manual counts were performed by three observers, who were allowed to adjust the image brightness to best facilitate counting accuracy. Images were collected as a Z-stack consisting of two to three planes of focus 0.5 $\upmu \hbox {m}$ apart per field in order to allow the observer to confirm the presence of Iba-1 positive cell bodies that were only partially in focus. The plane showing most cells in focus was used as the primary plane for counting. The observers used different visualization software during counting. Observer A used the Slidebook software (Intelligent Imaging Innovations, Denver, CO) paired with the microscope and Observers B and C used the Fiji distribution of ImageJ 2.1.0 for manual counting. Additionally, Observer A’s count was performed prior to the start of this project, and count markers were placed on images in close proximity to cell bodies for rapid total summation. Observer B and C placed counts within cell bodies to allow for later cell-level accuracy assessment. Microglia counts were normalized to area in cases where a part of the image area was unsuitable for cell detection, due to tissue damage or thickness irregularity (n = 3 images out of 62 total in study).

### Fluocell public dataset

**Figure 3:**
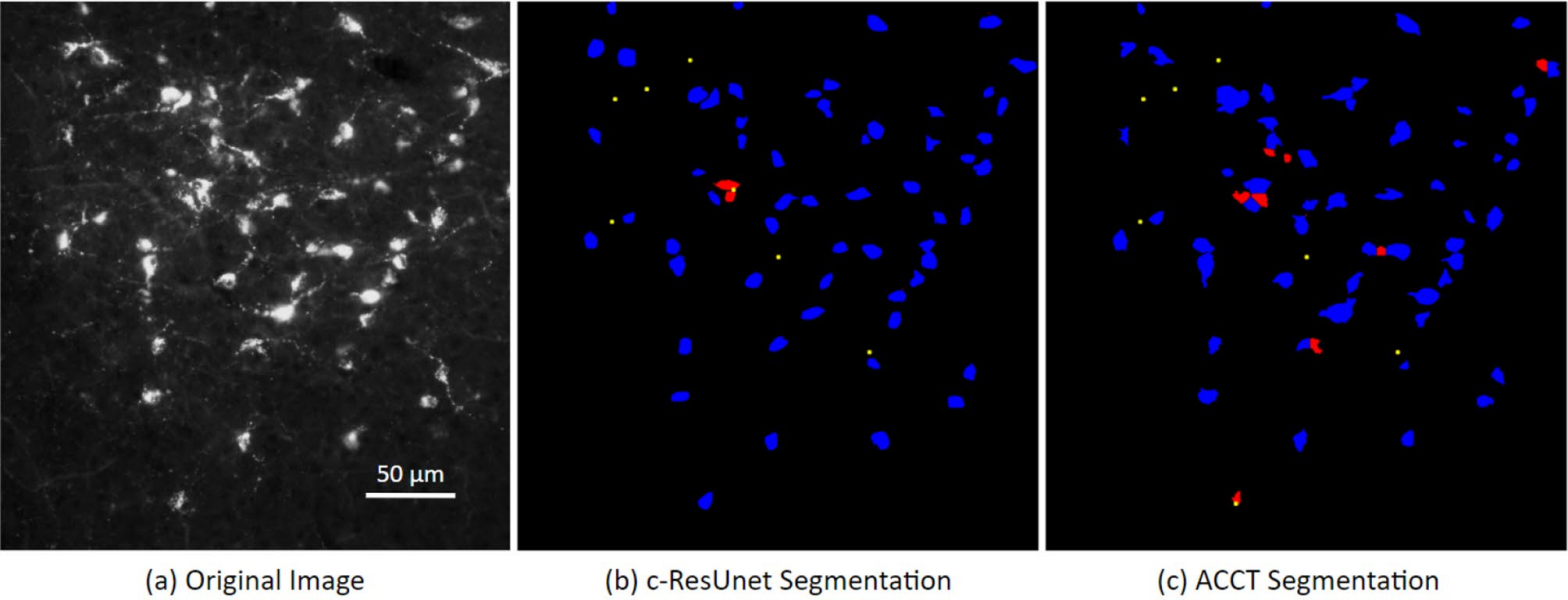
Image of fluorescence-labeled neurons before and after segmentation. A cropped image taken from the Fluocell dataset paired with cell segmentation (**a**). This depicts segmented cell objects from image $\hbox {MAR38S1C3R1}\_\hbox {DMR}\_20\_\hbox {o}$ reported in the publicly available Fluocell dataset segmented by c-ResUnet^10^ (**b**) and ACCT with classifierBayes3 (**c**). Resulting object segmentations are color-coded (blue = true positive, red = false positive, yellow = false negative). ACCT was set to filter out objects smaller than 250 pixels and greater than 5000 pixels to remove noise. We applied the watershed algorithm to this dataset. ACCT correctly identified 84.6% of the hand counts in this image and c-ResUnet 86.2%, while ACCT was correct with 86.9% of all predictions and c-ResUnet with 93.3%. Scale bar: 50 $\mu m$.

To further examine and develop the effectiveness of ACCT on a variety of data, we also performed a cell counting study using a publicly available image set^9,10^. The 283 1600x1200 pixel images were taken at 200x magnification of 35 $\upmu \hbox {m}$ thick slices of mouse brain tissue with neurons stained via a monosynaptic retrograde tracer (Cholera Toxin b, CTb). This tracer highlighted only neurons connected to the toxin injection site.

This dataset contains images with both high and low cell density, as well as varying amounts of noise and artifacts (Supplementary Fig. S2). We also observed that many images contain overlapping or touching cells. The Fluocell dataset presents different challenges when compared to our Iba-1 positive microglia dataset where cells are more evenly distributed and the number of cells per image is more consistent. A representative example of Fluocell data is shown in Fig. 3.

In the Fluocell analysis of this data, a subset of the images were manually counted by the authors, and the remaining images were counted via automatically thresholding^9^. Since we wish to compare ACCT to human placed cell counts as ground truth labels to assess performance of our tool versus human cell counting, we manually counted the entire 283 image dataset (one observer). This allows us to validate our tool against manual observer cell counts rather than another automatic process. In addition, the authors of the Fluocell dataset wrote their own automated cell counting program using a CNN approach named c-ResUnet which builds upon ResUNet^9^. Thus, we also compare the performance of ACCT versus c-ResUnet and Ilastik on the Fluocell dataset with our manual counts. We acknowledge that better classifiers may be possible to be generated for Ilastik and CellProfiler for our dataset, however we maintain that these programs lack the functionality for users to generate and assess multiple classifiers at large scale. Therefore, we generated fewer classifiers for these programs and selected the optimal classifier during training.

### Automatic counting methodology

ACCT is open source, available on GitHub at https://github.com/tkataras/Automatic-Cell-counting-with-TWS.git. Our machine learning classifier was built using the TWS plugin version 3.2.34 in ImageJ 2.1.0 included in the Fiji distribution^18^. In addition, the open source Python packages: scipy, pandas, numpy, matplotlib, imageio, and scikit-learn were used in ACCT^19–24^.

ACCT allows for the selection of several different types of machine learning approaches. Machine learning here refers to dynamic models trained on user specified input data to select cell pixels within an image. Users can also upload additional machine learning approaches compatible with Weka if desired. For this paper, we use an implementation of the Random Forest approach, called Fast Random Forest^4,25^. This is the default machine learning approach in TWS and the following default features were used:

- Gaussian blur
- Sobel filter
- Hessian
- Difference of Gaussian
- Membrane Projections
- Membrane thickness = 1
- Membrane patch size = 19
- Minimum sigma = 1.0
- Maximum sigma = 16.0

We additionally use a Bayesian Network model, which is also implemented in Weka^4^. This approach, called BayesNet, follows a Bayesian statistical model to determine the probability that observed features are conditionally dependent, or caused, by the object of interest^26^. For this study, we use the following parameters for Bayesian pixel classification, in addition to the above listed features:

- Variance
- Mean
- Minimum
- Maximum
- Median
- Anisotropic diffusion
- Bilateral
- Lipschitz
- Kuwahara
- Gabor
- Entropy
- Neighbors

#### Noise removal

During cell detection, small and large cellular processes or artifacts can be classified as cell bodies given similar enough appearance to cells. We address this noise by implementing a minimum and maximum cell object size parameter when counting cells. Thus, objects outside the specified size range are excluded from the automatic count. This range is empirically determined by observed cell bodies during model training and validation.

An additional challenge for cell detection is when two or more cells abut or overlap. This causes multiple cells to be identified as one large cell, so ACCT must separate these objects to increase accuracy. Thus, we optionally enable a watershed algorithm post pixel segmentation^27^. This algorithm is used to separate objects by contour, which allows for separated objects to be counted independently. We use the default implementation of the watershed algorithm provided in ImageJ^27^. We utilized the watershed segmentation strategy in the Fluocell dataset which had closely grouped cells, and compared ACCT’s performance with and without watershed to demonstrate the effect of the algorithm in Supplementary Fig. S3.

#### Cell body detection

The machine learning models in TWS generate a probability map for each image which is a representation of each pixel in the image as the probability that it is part of an object of interest. This probability is compared to a confidence threshold, which is the minimum probability a pixel must be to be considered part of an object. The user can set different threshold values, which affects how conservative or liberal the program will be in identifying objects. By default, ACCT starts at a threshold of 0.5 which can be modified by users through the user interface. Conventionally, stricter thresholds lead to fewer false positives but also fewer true positives. The inverse also holds with a more relaxed threshold identifying more true positives, but also more false positives. The performance of ACCT at various thresholds is represented visually on a Receiver Operator Characteristic (ROC) curve. However, some models usable with TWS in ACCT only give a binary zero or one for their confidence values which prevents generation of meaningful ROC curves.

### Iterative training and validation

Training on the Iba-1 microglia dataset was drawn from 10 randomly selected images not used in the counting analysis. These images were collected using the above described methods from mice distributed equally between experimental genotypes (Fig. 2). We note that ACCT is currently able to only handle single-channel images, rather than multi-channel images. Incremental adjustments to training data and resulting changing pixel classification was observed in real time and the classifiers were saved sequentially.

To avoid overtraining, classifiers are updated a few pixels at a time with new training data and the updated pixel segmentations on training data are observed immediately in TWS. Subsequent training data is selected to address areas of incorrect segmentation. We continue this over successive iterations of classifiers, saving a version of the classifier after each addition of training data. This iterative classifier creation scheme continues until the classifier does not appear to be improving on the data. ACCT then performs accuracy assessment on validation data and ground truth markers, accounting for experimental conditions, to help the user select the classifier iteration with the greatest accuracy and consistency across the validation dataset.

This strategy was applied to create multiple sequential classifiers which were subsequently applied to the validation dataset (Fig. 1, Supplementary Fig. S1). Ultimately, 25 sequential classifiers were trained on the Iba-1 microglia data. The Iba-1 microglia validation dataset was comprised of 10 images (Resting n = 5, Activated n = 5) from the main dataset which was then excluded from all further analyses. Cell body location specific count markers were placed in these images by Observer B, and performance was calculated via precision, recall, F1 score, accuracy, as well as a Student’s T-Test of differential accuracy between the Resting and Activated images. ACCT is also able to perform ANOVA calculations for further analyses including more than two experimental groups.

In the Fluocell data, 10 training images were selected from the dataset to represent the variety of segmentation challenges within the dataset: highly variable intensity, highly variable cell density, overlapping cell images, and images with non-cell artifacts. The validation dataset was made with a different set of 10 images selected to represent a similar distribution of challenges.

### Classifier selection

True positive scores for each image are determined by the localization of manual count markers which are checked against the pixel location values of each object for the automatic counting process. False positives for each image are represented as the total number of automatically generated cell objects that do not contain a single manual count. False negatives for each image are determined by the total number of manual count markers that are not contained inside of an automatically generated object by the program, plus the number of manual count markers inside of a single cell object in excess of one, indicating insufficient object separation. As we assess accuracy based on cell location and do not differentiate between background pixels, ACCT does not include determination of true negative cell locations. This methodology was used in Morelli et al.^9^, and we calculate accuracy as $\frac{TP}{TP + FP + FN}.$

We assess the performance of our classifiers using measures of precision, recall, F1 score, and accuracy. Precision is the proportion of automatic counts that are correct based on manually placed markers, and recall is the proportion of the total manually placed cell markers that were successfully identified by the automatic count. The F1 score is the harmonic average of precision and recall. The accuracy is assessed specifically as the number of true positive cell counts as a proportion of all manual and automatic counts, including false negatives. Multiple classifiers can be evaluated through automatically calculated statistical analysis. Statistical measures, such as mean absolute error (MAE), are additionally calculated through ACCT to evaluate the performance of different classifiers. We used these statistics to assess the performance of ACCT against other automatic cell counting tools based on these metrics.

**Figure 4:**
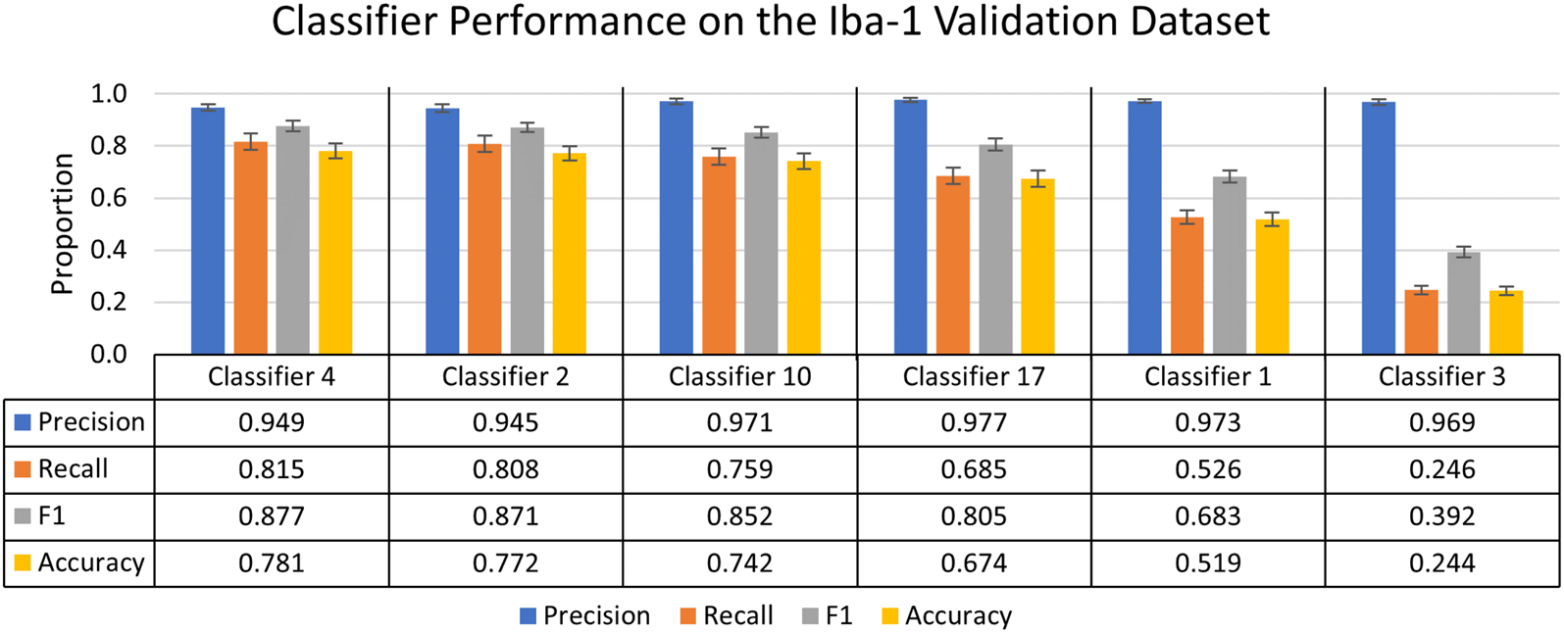
Summary of individual classifier performance on the Iba-1 microglia dataset during the validation stage. A chart of the most and least accurate three incrementally trained classifiers, ranked by F1 score of 25 trained classifiers (n = 10 images). Error bars represent standard error of the mean on calculated performance statistics from each image, where the statistic itself is calculated from total cells in the dataset. The parameters used in ACCT: 0.5 threshold, 50 minimum pixel size, and 1,000 maximum pixel size.

This statistical information is shown in Figs. 4 and 9. Figure 4 is a subset of the full data which can be found in Supplementary Table 1. Figure 9 shows selected accuracy statistics at different confidence thresholds. Classifier selection via the best F1 score or different weighting of precision and recall, are all valid metrics for selecting a classifier. However, for this study we have selected the classifier based on the highest F1 score.

### Experimental dataset analysis

As the next step, the selected classifier is applied to the experimental dataset of images. This experimental dataset excludes images used in training and validation. The automated counting methodology is repeated in this analysis and reports the total number of cells counted in addition to other statistical information per image. Morphological information about each identified cell is reported by ACCT to users. An example of this can be found in Supplementary Table 2, which lists some of the reported morphological information generated from analysis.

### Selected classifier performance audit

**Figure 5:**
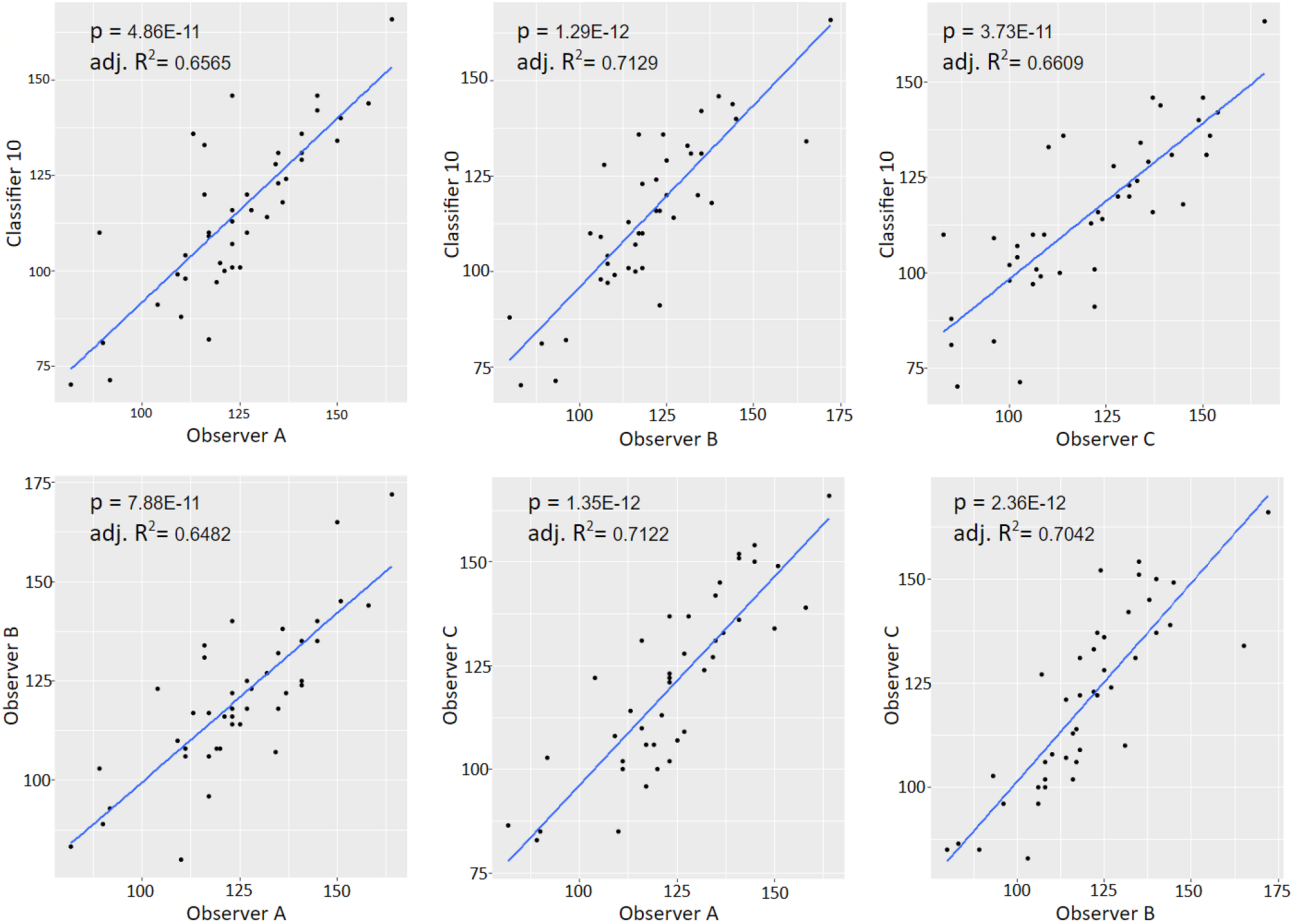
Correlation analysis of microglial density scatter plots with regression lines for all correlative comparisons between observer counts and the automatic count from classifier 10 for the experimental dataset. All relationships showed significant, positive overall correlations (p and Adj. $R^{2}$ = values included in the figures; $\hbox {n}=42$).

We additionally have the option for users to audit the performance of their selected classifier. The audit requires further manual counting and is identical to how we assess the performance of classifiers during the validation stage. This step is intended to determine how similarly the classifier performed on the experimental dataset compared to the validation set in situations where all images have not been manually counted. The audit can be performed using a subset of the experimental dataset, or even the whole dataset, if the user chooses to complete an entire manual count to assess model accuracy. These images are known as the audit set. We randomly selected 5 images each of Activated and Resting microglia experimental images for the Iba-1 audit set. An audit of the Fluocell data was performed on a sample of 10 images from the Fluocell experimental dataset (Fig. 5).

## Results

### Microglia density

#### Iba-1 microglia dataset

**Figure 6:**
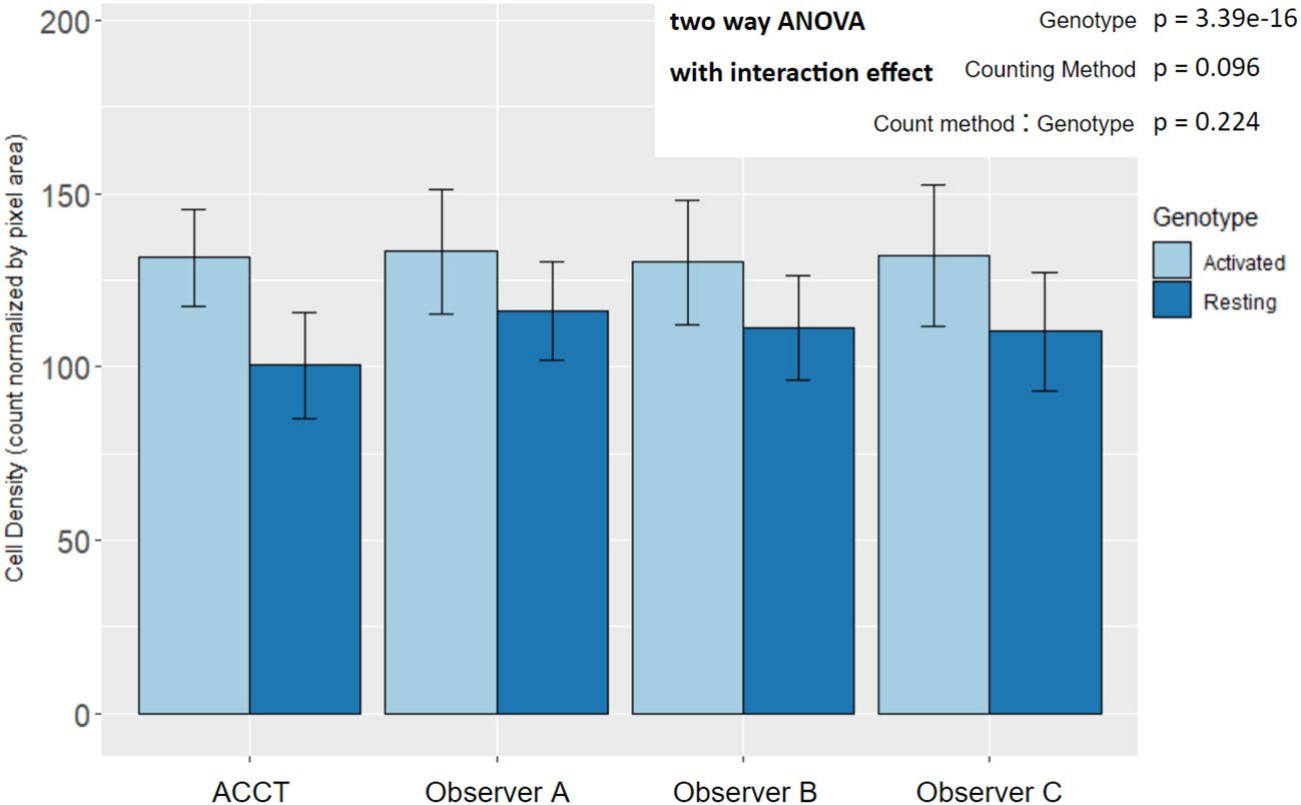
Mean microglia density by experimental genotype in manual and automated counts. All counting methods found an increase in microglia density in Activated microglia images by two way ANOVA with interaction effect and Tukey HSD post-hoc analysis. The difference between counting method and the interaction effect did not display statistical significance. (Genotype: p = $3.39E^{-16}$; Counting Method: p = 0.096; Genotype:Counting Method: p = 0.224; Resting n = 22, Activated n = 20). There were not significant differences between the automatic count and Observer B and C. However, the ACCT count density trended lower overall than Observer A’s (p = 0.0599; Resting n = 22, Activated n = 20). This suggests that classifier 10 may have excluded some faintly stained or not well-in-focus cells in the Resting group images that Observer A did count. Error bars represent standard deviation.

All statistical tests on Iba-1 microglia images include all images except those used for training and validation (Resting n = 22, Activated n = 20). We report classifier 10’s automatic count instead of classifier 4 because classifier 10 provided the maximum performance on the experimental dataset and audit set that we observed. The significant increase in microglia density in images of gp120 positive (Activated) mice was consistent across the dataset via two way ANOVA ($\hbox {p} = 3.39E^{-16}$; Resting n = 22, Activated n = 20) (Fig. 6).

Accounting for genotype variance within the dataset no significant differences were found between the ACCT count and Observers (Observer A p = 0.060; Observer B p = 0.514; Observer C p= 0.440). Additionally, the per image microglia density demonstrated significant correlations among the automatic count and all observers with stronger correlations in Resting microglia images (Fig. 5).

The following represents the total cell counts:

- Validation: Observer A/B 1263/1380 cells over 10 images.
- Experimental Dataset: Observer A/B/C 5158/5035/5056 cells over 42 images.
- Audit Set: Observer A/B/C 1239/1207/1262 cells over 10 images.

### Precision and recall

#### Iba-1 microglia dataset

**Figure 7:**
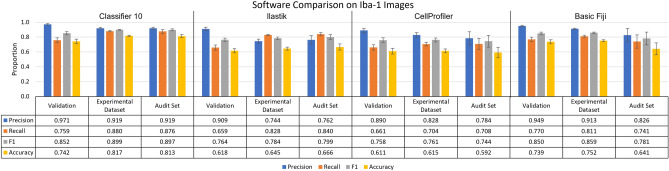
ACCT vs Ilastik vs CellProfiler vs Basic Fiji on images of Iba-1 positive microglia. The performance of ACCT classifier 10 versus Ilastik, CellProfiler, and basic use of Fiji tools. The audit set is a selection of 10 images, which is equal to the size of the validation image set, chosen from the experimental dataset and evenly distributed between experimental groups. Error bars represent standard error of the mean on calculated performance statistics from each image, where the statistic itself is calculated from total cells in the dataset. The parameters used by ACCT in this analysis were: a 0.5 threshold, 50 minimum pixel size, 1,000 maximum pixel size for objects.

The overall precision and recall achieved by the TWS methodology were similar in the validation dataset, experimental dataset, and audit dataset with overall accuracy and F1 increased in the experimental dataset compared to validation as shown for classifier 10 in Fig. 7. However, within the experimental dataset, the TWS classifier was more conservative in the Resting images compared to Observer B’s manual counts, with the automatic count having higher precision in images of Resting than Activated samples (Precision p = 0.007477) (Fig. 7). When compared to Ilastik and CellProfiler, ACCT had a similar, and slightly stronger performance than both tools in each set of Iba-1 images, with Ilastik slightly outperforming CellProfiler. We additionally compared these tools against basic functionality that users can manually select in Fiji, to illustrate how ACCT builds upon existing Fiji functionality. We used the subtract background with rolling ball, adjust threshold tools in Fiji for this analysis, with background subtraction at 25 pixel area and pixel intensity threshold of 90. Without applying minimum and maximum object size this analysis resulted in near 0 precision, thus we used a 50 minimum and 1000 maximum pixel size as in the other tools. Classifier 10 outperforms the basic Fiji tools in most metrics except for precision. The basic Fiji application also narrowly outperforms Ilastik and CellProfiler in most metrics in the Iba-1 images.

#### Fluocell dataset

**Figure 8:**
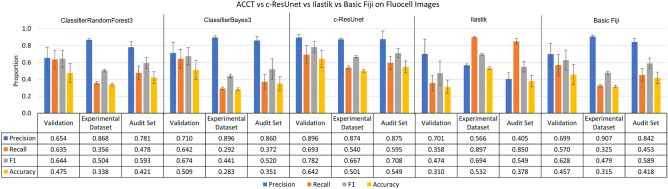
ACCT vs c-ResUnet vs Ilastik vs Basic Fiji on the Fluocell dataset. ClassifierRandomForest3 and ClassifierBayes3 are the third trained iterations of a Fast Random Forest and BayesNet model in ACCT, respectively. The three tools’ automatic counts of the Fluocell images and basic use of Fiji tools are compared to our manual count of the Fluocell dataset. Error bars represent standard error of the mean on calculated performance statistics from each image, where the statistic itself is calculated from total cells in the dataset. The audit set is a selection of 10 images, which is equal to the size of the validation image set, chosen from the experimental dataset. The parameters used are: a 0.5 threshold, 250 minimum pixel size, 5000 maximum pixel size, and with the watershed algorithm applied.

In contrast to the Iba-1 microglia dataset, the Fluocell dataset does not compare two different experimental conditions. All Fluocell statistical tests include all Fluocell images except those used in training and validation (n = 263). In Fig. 8, we compared the performance of the Fast Random Forest and BayesNet models implemented within ACCT versus c-ResUnet, Ilastik and basic Fiji usage^9,12^. Figure 8 shows ClassifierRandomForest3 outperforming BayesNet on most statistical metrics. Additionally, the c-ResUnet model outperformed on most metrics compared to these two classifiers. In contrast to the other tools, Ilastik has much greater recall than precision in the experimental and audit datasets, with ACCT and c-ResUnet outperforming on precision. However, Ilastik has the greatest F1 score in the experimental dataset. Basic Fiji methodology struggled in much the same way as the other classifiers, with higher precision and low recall, it provided comparable accuracy to the ACCT classifiers on the experimental dataset. For basic Fiji we used background subtraction at 50 pixel area and pixel intensity threshold of 70. In the context of this dataset, the following represents the total cell count:

- Validation: 137 cells over 10 images.
- Experimental Dataset: 3307 cells over 263 images.
- Audit Set: 247 cells over 10 images.

### Receiver operator characteristic

**Figure 9:**
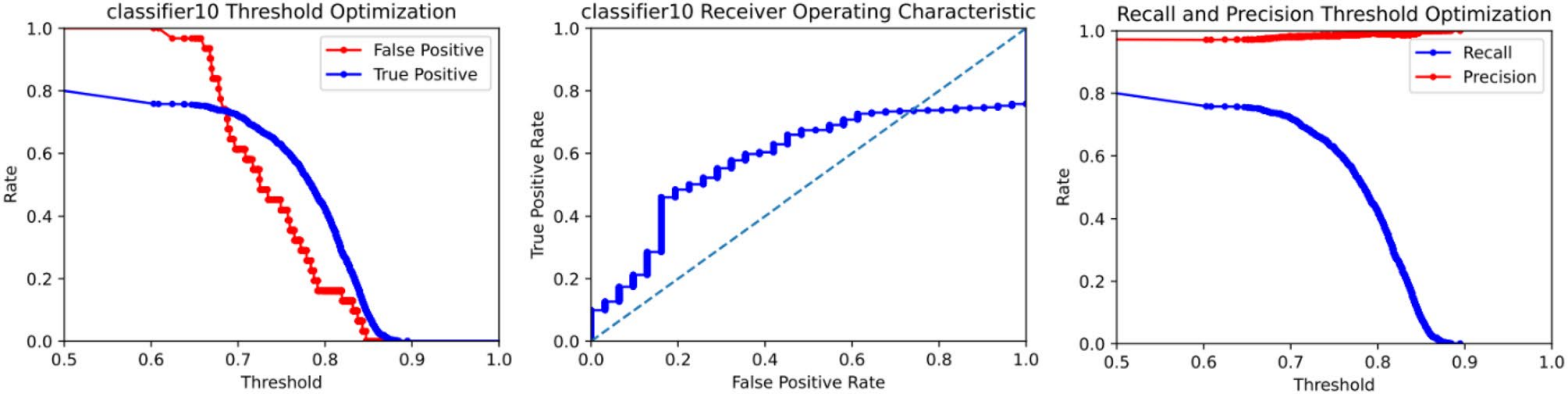
An ROC curve generated by ACCT following the validation stage on Iba-1 microglia images. It depicts the false positive, true positive, recall, and precision rates of classifier 10 at different confidence thresholds on the Iba-1 stained microglia images. Objects were filtered to a minimum size of 50 pixels and a maximum size of 1000 pixels. The watershed algorithm was not applied to the Iba-1 dataset, due to consistent cell separation in the sample tissues.

ACCT automatically generates ROC curves for each trained classifier. This visualizes the tradeoffs between precision and recall as well as the true positive rate and the false positive rate. Figure 9 demonstrates a ROC curve of ACCT classifier 10 applied to the Iba-1 microglia dataset. The threshold represents the required probability from the classifier to determine if a pixel will be designated as a cell pixel. The data represented in these graphs were generated using the scikit-learn Python library which performed statistical analysis^24^.

Figure 9 demonstrates the tradeoff in which the false positive rate decreases at a faster rate than the true positive rate when a higher threshold is applied. For example, increasing the threshold for pixel segmentation in the Iba-1 dataset reduced the false positive rate compared to the default of 0.5. In this study, the 0.5 threshold was used for reported calculations, as overall accuracy did not increase due to a decrease in true positive cell identifications.

## Discussion

ACCT is a step towards more accessible computation tools for cell counting and image segmentation. The main current advantage of this strategy is the shorter time required to train and apply the automatic counting strategy compared to manually counting each image. Our study demonstrates the general applicability of this tool to quickly explore large amounts of data.

The training process is critical for the success of this automatic cell counting methodology and relies on a researcher’s specific knowledge of their imaged cell type. Each image set comes with its own unique set of challenges due to variability in cell and media characteristics, so providing accurate training data requires a firm and consistent understanding of the images in question. ACCT is able to help users adjust for these features in their images. Users can select specific features to be analyzed in their selected machine learning models to better represent their image data. Since every user has different data, this added flexibility improves the ability for ACCT to analyze users-specific images.

The results demonstrate that these ACCT performs strongest on precision, indicating that most of the ’called’ cells were real cells. Recall tends to be substantially lower than precision, leading to decreased F1 scores and accuracy in all ACCT classifiers tested on these datasets. This indicates that these models tend to be more conservative than expert manual counts. However, the results indicate that when models classify an object as a cell, they tend to be correct based on the high precision.

Additionally, microglia density analysis in Fig. 6 and in Fig. 5 demonstrate that ACCT counts cells similarly to expert observers. Counts of all observers identified a similar mean difference in microglia density between experimental genotypes. ACCT correlates strongly with human cell counting results and can replicate the difference between experimental conditions similar to manual counting. Thus, it is a useful tool for image analysis between multiple experimental conditions.

We acknowledge that in the future more accurate automatic cell counting tools are likely to evolve from ACCT or other software packages. However, currently ACCT shows strong performance while being a more accessible tool to researchers than all approaches which require large computer networks or computing clusters. In some cases ACCT outperforms other cell counting tools, but not in every dataset. However, users may find the accessibility in the ImageJ environment and speed of ACCT worth trading for the slight loss in performance in some image data sets.

In terms of computational power, all work was performed on commercially available consumer laptops such as a Dell Inspiron 15-7559 (released Feb. 2017). Other automatic cell counting tools are often designed to make use of large computational resources. For example, Morelli et al. used 4 V100 GPUs to process their CNN approach^9^. CNN based tools recommend using a cluster, or network of multiple computers, which allows access to greater computational power. However, computer clusters are not available for all researchers and they additionally may require knowledge of command lines for effective utilization. ACCT is not limited by substantial computer specifications, additionally it is more accessible to less command line-oriented researchers.

Reproducible results are a major concern in scientific research, and ensuring reproducibility via manual cell counting can be costly and time consuming. Since ACCT stores classifiers as single model files, they are easy to share and download. Thus, researchers can share reproducible results and statistical analysis of a cell counting study by sharing the model file and set of analyzed images. Since analysis generated by ACCT is stored as files editable in Excel, it is easy for users to share and communicate their results.

Building ACCT around the graphical interface implemented by TWS broadens usability by providing infrastructure for quantitative classifier validation and application in a full experimental context onto the flexible and intuitive training apparatus^4^. Since ACCT makes use of existing tools for cell imaging analysis such as TWS, researchers familiar with the program should find it easier to learn how to use ACCT as well.

ACCT includes accessible documentation, with an instruction manual that explains the program’s function and usage found on its GitHub page. Documentation is important for users to understand and learn how to use software tools. Many other software tools document their components’ functions inside of the tool itself, requiring users to navigate code to understand and use the tool. This is avoided by having detailed instructions written on the website for ACCT which explain the use of the tool without ever requiring the users to manually access the code itself.

ACCT could also be applied more generally to image segmentation problems. While the focus of our study is on cell counting in the context of neuroscience, so long as an image has object characteristics that can be separated from its background, ACCT is able to quantify the objects. However, more complex object shapes and less distinctive backgrounds may require selecting more complex models than demonstrated in this study. We provide a simple example of this in Supplementary Fig. S4 using the Fast Random Forest model, which is an image segmented for buildings against a field^28^. While not as distinct as cells, it demonstrates that ACCT is applicable beyond the biological context. Overall, ACCT should greatly increase the accessibility of automatic analysis involving cell counting for a wide audience in neuroscience research and beyond.

## Data Availability

The Iba-1 dataset, its analysis, and the Fluocell dataset analysis during the current study is available in the ACCT-Data-Repository, https://github.com/tkataras/ACCT-Data-Repository.git. The Fluocell dataset analyzed is publicly available as published in http://amsacta.unibo.it/6706/.

## Supplementary lnformation

Supplementary Figure 1. Supplementary Figure 2. Supplementary Figure 3. Supplementary Figure 4. Supplementary Table 1. Supplementary Table 2.

## Code availability

ACCT can be accessed and downloaded from GitHub at https://github.com/tkataras/Automatic-Cell-counting-with-TWS.git.
