# Supplementary figures and images for: ACCT is a fast and accessible automatic cell counting tool using machine learning for 2D image segmentation
Source: Sci Rep. 2023 May 22;13:8213. doi: 10.1038/s41598-023-34943-w (PMC10202925; doi:10.1038/s41598-023-34943-w)

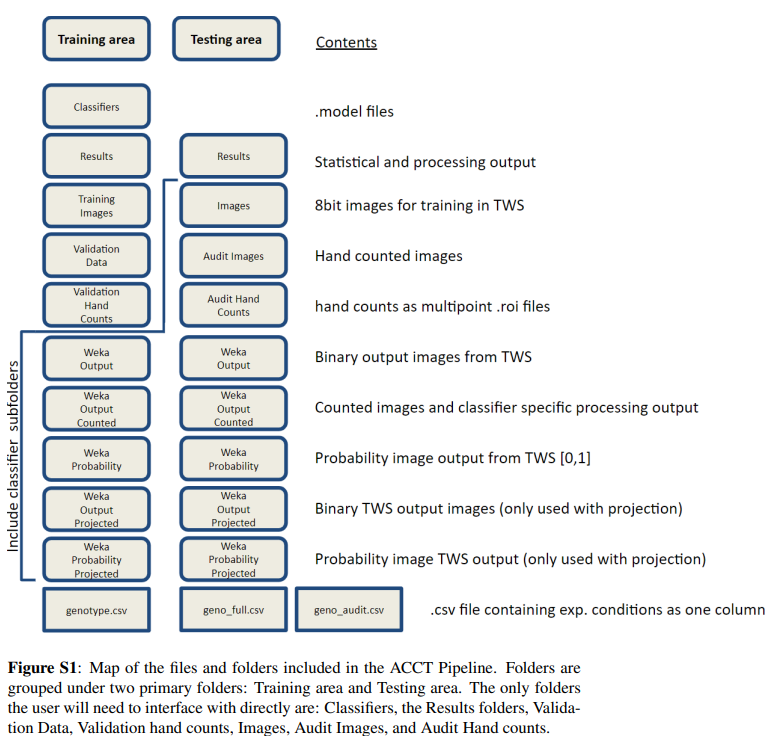

Supplement: Supplementary file 1 — Supplementary Figure 1. [file 41598_2023_34943_MOESM1_ESM.jpg]

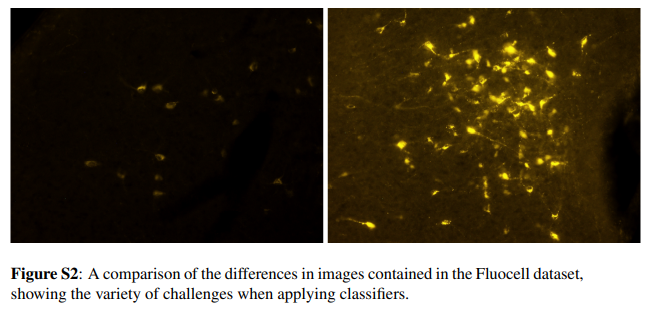

Supplement: Supplementary file 2 — Supplementary Figure 2. [file 41598_2023_34943_MOESM2_ESM.jpg]

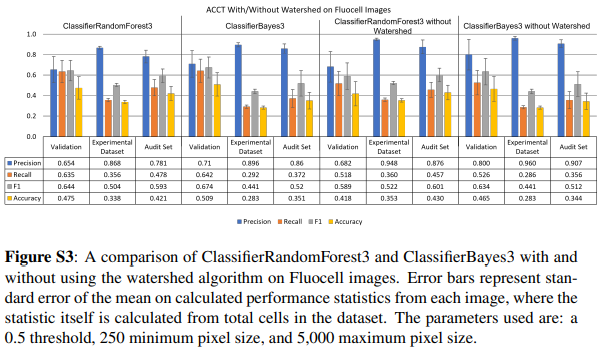

Supplement: Supplementary file 3 — Supplementary Figure 3. [file 41598_2023_34943_MOESM3_ESM.png]

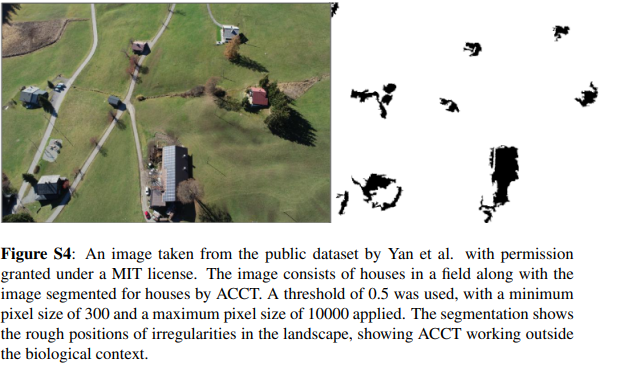

Supplement: Supplementary file 4 — Supplementary Figure 4. [file 41598_2023_34943_MOESM4_ESM.png]
